# Angiomyomatous hamartoma of the inguinal lymph nodes with localized lymphedema presenting as a soft subcutaneous mass

**DOI:** 10.1016/j.jdcr.2022.07.025

**Published:** 2022-07-31

**Authors:** Ryoma Honda, Kazuyasu Fujii, Masanori Nakajo, Takuro Kanekura

**Affiliations:** a Department of Dermatology, Kagoshima University Graduate School of Medical and Dental Sciences, Kagoshima, Japan; b Department of Radiology, Kagoshima University Graduate School of Medical and Dental Sciences, Kagoshima, Japan

**Keywords:** angiomyomatous hamartoma, lymphedema, lymph nodes, magnetic resonance imaging, AMH-LNs, angiomyomatous hamartoma of the lymph nodes, MRI, magnetic resonance imaging

## Introduction

Angiomyomatous hamartoma of the lymph nodes (AMH-LNs) usually develops in the inguinal or femoral lymph nodes. Although its pathologic findings have been well described in the literature, there have been few reports on detailed clinical and imaging features of AMH-LNs. Moreover, most of these reported cases required excisional biopsy to make a precise diagnosis. Herein, we report a case of AMH-LNs with localized lymphedema manifesting as a subcutaneous mass. In this case, we also describe in detail the clinical and imaging findings to help dermatologists establish an accurate diagnosis without unnecessary surgical intervention.

## Case report

**Fig 1:**
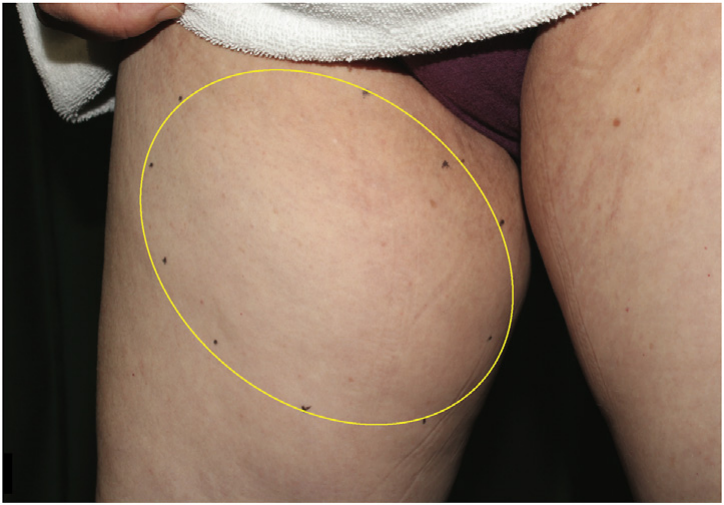
Clinical findings of the patient. A subcutaneous soft mass is observed in the patient’s right femur (*yellow oval*).

**Fig 2:**
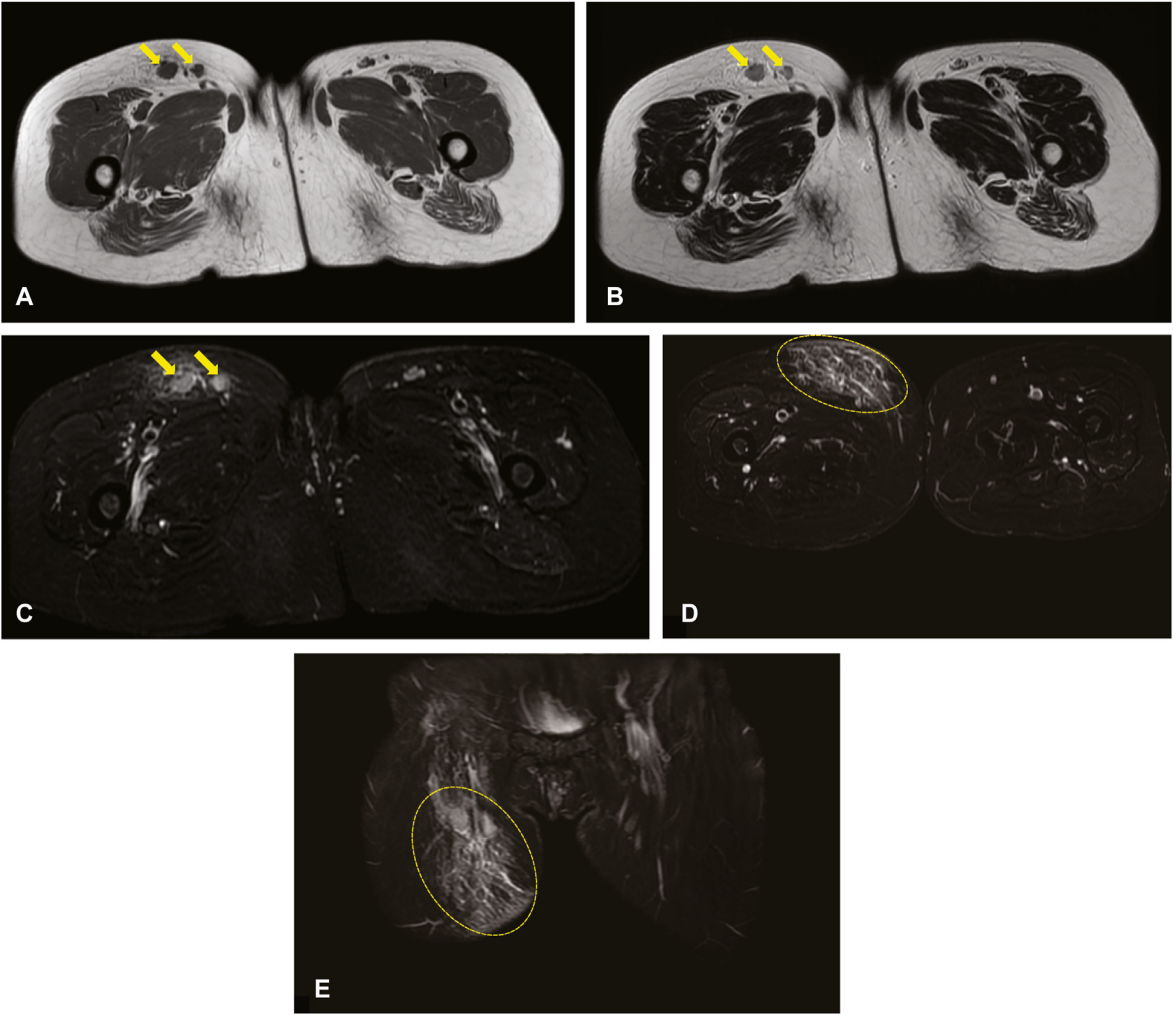
Imaging findings of the patient. Magnetic resonance imaging (MRI) reveals several lymph nodes in the right inguinal region. **A,** Transverse T1-weighted magnetic resonance imaging shows a homogeneous tumor with high signal intensity *(yellow arrows*). **B,** Transverse T2-weighted magnetic resonance imaging shows a heterogeneous tumor with high signal intensity (*yellow arrows*). **C,** The tumor region of interest is delineated on a diffusion-weighted image (*yellow arrows*) and (**D**) mirrored to an apparent diffusion coefficient map (*yellow oval*), which is derived using b-values of 0 and 1000 s/mm^2^. **E,** The corresponding apparent diffusion coefficient histogram of the whole tumor volume demonstrates a rightward shift (*yellow oval*) (apparent diffusion coefficient values [31,023 mm^2^/s]: mean, 1.76; 10th percentile, 1.33; 25th percentile, 1.51; 50th percentile, 1.75; 75th percentile, 2.02; 90th percentile, 2.20, Coefficient of Variation; 0.19; skewness; −0.05).

**Fig 3:**
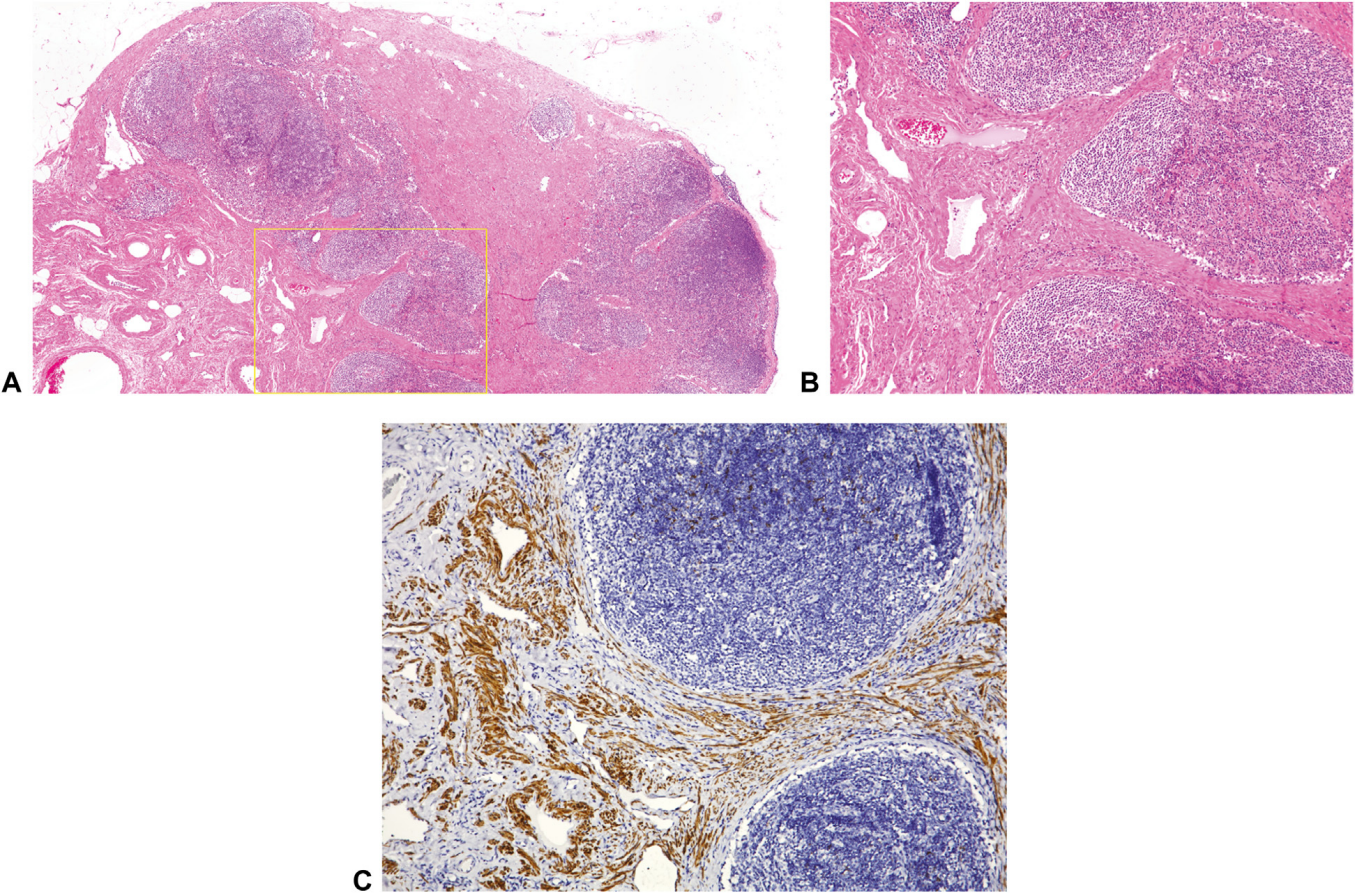
Histopathologic and immunohistochemical findings of the patient. **A** and **B,** Lymph nodal parenchyma is replaced partially by irregularly distributed thick-walled vessels and smooth muscle cells in a dense fibro-collagenous stroma. **C,** Using immunohistochemical staining, the stromal cells are positive for desmin (*brown*). **A** and **B,** Hematoxylin-eosin stain; **C,** immunohistochemical stain; original magnification: **A,** ×12.5; **B,** ×40; **C,** ×100).

A 63-year-old woman without any remarkable medical or family health history was referred to our clinic with a complaint of a right femoral subcutaneous soft mass measuring 10 cm × 10 cm in size (Fig 1). Ultrasonography revealed a subcutaneous mass, including a few hypoechoic lesions in the subcutaneous tissue. Magnetic resonance imaging (MRI) revealed several swollen lymph nodes in the right inguinal region. Lymph nodes showed homogenous iso-signal intensity on transverse T1-weighted imaging (Fig 2, *A*) and slightly high heterogeneous signal intensity on T2-weighted imaging (Fig 2, *B*). Transverse fat-suppressed T2-weighted MRI (Fig 2, *C*) and an apparent diffusion coefficient map (Fig 2, *D* and *E*) showed slightly swollen lymph nodes surrounded by edema. Excisional biopsy of 1 of the lymph nodes from the mass demonstrated partial replacement of lymph node parenchyma by proliferated thick-walled vessels distributed in the hilum, extending into the lymph node cortex (Fig 3, *A* and *B*). Irregularly arranged smooth muscle cells were observed in the dense fibrocollagenous stroma. Immunohistochemical examination revealed that the stromal cells were positive for desmin (Fig 3, *C*). On the basis of these findings, we established a diagnosis of AMH-LNs with localized lymphedema. During the 4-month follow-up period after diagnosis and without any treatment, the mass was not enlarged.

## Discussion

Angiomyomatous hamartoma is histologically characterized by the partial replacement of normal nodal parenchyma by disorganized blood vessels and smooth muscle cells, with or without adipose tissue within a fibrous stroma.^1^ The exact pathogenesis of AMH-LNs remains unclear. However, some reports have indicated that impairment of lymphatic flow might be involved in the pathogenesis of AMH-LNs.^2^, ^3^, ^4^ Remarkable lymphedema, observed in this case as a giant soft subcutaneous mass, indicated the simultaneous disturbance of numerous lymphatic circulations.

To date, more than 60 cases of AMH-LNs have been reported.^5^, ^6^, ^7^, ^8^, ^9^ Most cases of AMH-LNs reported so far had solitary lesions. In contrast, several enlarged lymph nodes, similar to the excised lymph node, were observed in the right inguinal area on MRI in the present case. AMH-LNs with multiple lesions is extremely rare. Our review of the literature uncovered only 1 case of AMH-LNs comprising 2 lesions within a mass.^3^

Most reported cases of AMH-LNs were presented by pathologists from a histopathologic point of view. In addition, few previous reports have provided a detailed clinical description of AMH-LNs with imaging findings. Of note, to our knowledge, although there has been only 1 report describing the clinical presentation of AMH-LNs,^10^ there has been no report presenting a subcutaneous mass, as in this present case. MRI findings of AMH-LNs were described in only 3 previous reports, which demonstrated a well-circumscribed solitary nodule with heterogeneous signal intensity.^4^^,^^5^^,^^10^ In the present case, lymph nodes showed a slightly high heterogeneous signal intensity on T2-weighted imaging, similar to previously reported radiologic features. However, on transverse T1-weighted imaging, these lymph nodes showed homogenous iso-signal intensity. Moreover, the patient exhibited localized lymphedema on transverse fat-suppressed T2-weighted MRI and an apparent diffusion coefficient map. These findings have not been previously indicated.

This report describes important clinical and imaging features of AMH-LNs and lymphedema. It is difficult to make a diagnosis of AMH-LNs before excisional biopsy because of its rarity and close appearance to other soft-tissue tumors, such as lipoma, lipomatosis, liposarcoma, angiosarcoma, and fibroblastic neoplasm. Furthermore, AMH-LNs needs to be differentiated from other diagnoses that cause localized lymphedema (pseudosarcoma in patients with obesity, massive blunt trauma, lymphadenectomy, and myxedema). Because unnecessary surgical treatment could be avoided if diagnosis of this rare benign entity could be made by other diagnostic criteria, an accumulation of reports that describe detailed clinical and imaging features of AMH-LNs is required to enable a precise nonsurgical diagnosis of AMH-LNs. AMH-LNs is still uncommon in dermatology. Thus, dermatologists should be aware of this rare disorder and recognize localized lymphedema, especially in the evaluation of soft subcutaneous masses, to avoid unnecessary surgical treatment.

## Conflicts of interest

None disclosed.
